# Insights into Neuroinflammation in Parkinson's Disease: From Biomarkers to Anti-Inflammatory Based Therapies

**DOI:** 10.1155/2015/628192

**Published:** 2015-07-29

**Authors:** Natália Pessoa Rocha, Aline Silva de Miranda, Antônio Lúcio Teixeira

**Affiliations:** Laboratório Interdisciplinar de Investigação Médica, Faculdade de Medicina, Universidade Federal de Minas Gerais, Avenida Professor Alfredo Balena 190, Sala 281, 30130-100, Belo Horizonte, MG, Brazil

## Abstract

Parkinson's disease (PD) is the second most common neurodegenerative disorder worldwide, being characterized by the progressive loss of dopaminergic neurons in the substantia nigra pars compacta. Among several putative factors that may contribute to PD pathogenesis, inflammatory mechanisms may play a pivotal role. The involvement of microglial activation as well as of brain and peripheral immune mediators in PD pathophysiology has been reported by clinical and experimental studies. These inflammatory biomarkers evaluated by imaging techniques and/or by biological sample analysis have become valuable tools for PD diagnosis and prognosis. Regardless of the significant increase in the number of people suffering from PD, there are still no established disease-modifying or neuroprotective therapies for it. There is growing evidence of protective effect of anti-inflammatory drugs on PD development. Herein, we reviewed the current literature regarding the central nervous system and peripheral immune biomarkers in PD and advances in diagnostic and prognostic tools as well as the neuroprotective effects of anti-inflammatory therapies.

## 1. Introduction

Parkinson's disease (PD) is the second most common neurodegenerative disorder worldwide. The major pathological findings in PD are the progressive loss of dopaminergic neurons in the substantia nigra pars compacta and the presence of intraneuronal inclusions of the protein *α*-synuclein (known as Lewy bodies) [1]. Neuronal death in the substantia nigra results in dopamine deficit at the striatum and, as an outcome, the clinical hallmarks of Parkinsonism: bradykinesia, rigidity, resting tremor, and postural instability. PD diagnosis, which is essentially clinical, is based on the diagnosis of Parkinsonian syndrome and the exclusion of other causes of Parkinsonism [2]. Good response to levodopa and asymmetry of motor symptoms support the diagnosis. Although PD is traditionally regarded as a movement disorder, motor symptoms may be heralded or accompanied by several nonmotor symptoms, such as hyposmia, constipation, neuropsychiatric, and sleep disorders [3].

**Figure 1:**
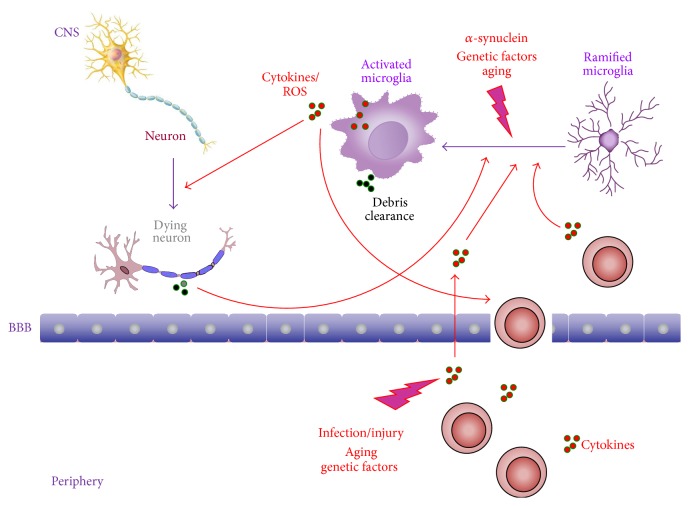
Inflammatory pathways in Parkinson's disease. An acute insult to CNS (e.g., *α*-synuclein aggregates) triggers the activation of microglia with changes in their morphofunctional characteristics, increased proliferation and release of inflammatory mediators (e.g., cytokines and ROS). Inflammatory molecules can induce the recruitment of peripheral leukocytes into the CNS. This neuroinflammatory process can be regarded as beneficial for neuronal tissue since it promotes clearance of cell debris. Conversely, inflammatory mediators do not modulate only immune cells but also act on neurons, contributing to neurodegeneration. Neuronal death further activates inflammatory mechanisms, resulting in a vicious cycle of inflammation and neuronal death. Systemic inflammation due to infection or peripheral injury can exacerbate symptoms and promote neuronal damage in PD. Leukocytes secrete proinflammatory cytokines which can affect the brain by several routes, including action on endothelial cells and leakage through damaged BBB. These cytokines induce self-synthesis and the synthesis of other cytokines, which can then stimulate microglia to secrete chronically inflammatory mediators, maintaining neuroinflammation and, as a consequence, slow and progressive neuronal death. Genetic and aging factors might contribute to this process. BBB: blood-brain barrier; CNS: central nervous system; PD: Parkinson's disease, ROS: reactive oxygen species.

PD was first described in 1817 [4], and despite the well-characterized pathological features, the cause of neuronal death in PD remains a matter of debate. Among several putative factors that may contribute to PD pathogenesis, inflammatory mechanisms may play an important role. For instance, microglial activation is associated with dopaminergic neuronal loss, which suggests that neuroinflammatory reaction may contribute to the progressive degenerative process. Moreover, it has been reported that the protein *α*-synuclein has an important role in the initiation and maintenance of inflammation in PD (see Figure 1) [5].

A recent meta-analysis revealed an overall prevalence of PD of 315 per 100,000 individuals. Prevalence of PD increases steadily with age, raising from 428 per 100,000 in individuals for the age group of 60 to 69 years, to 1,903 per 100,000 individuals for the group of 80 years or older [6]. Overall worldwide incidence of PD is estimated in 36.5 per 100,000 person-years for females and 65.5 per 100,000 person-years among males [7]. Most countries are facing marked demographic changes, with progressively larger proportion of their populations entering old age. PD affects predominantly the elderly, being a disease worthy of concern, since the causes are still unknown and the treatment is palliative and merely symptomatic. Levodopa, the first breakthrough in the treatment of PD, is still the most effective drug for the motor symptoms of the disease. In certain instances, other medications such as monoamine oxidase type B inhibitors, anticholinergics, and dopamine agonists may be initiated first to prevent levodopa-related motor complications [8]. Although the number of people suffering from PD rises significantly year by year, there are no established disease-modifying or neuroprotective therapies for PD. In this scenario, in the present review, we discuss current evidence regarding the contribution of immune dysfunction and/or inflammation in PD, advances in recent image techniques as valuable tolls for PD diagnosis and progression, and the perspectives of anti-inflammatory based therapies (data are summarized in Table 1).

## 2. Neuroinflammation in PD: Lessons from* Post-Mortem* and Neuroimaging Studies

### 2.1. Microglial Activation Role in PD

The first evidence of inflammation involvement in PD was derived from James Parkinson's report on the first clinical and pathological description of the disease in the early nineteenth century [4]. More direct evidence was provided much later in the twentieth century from systematic* post-mortem* analysis of the brain of PD patients [9]. Based on morphological features and immunohistochemical staining against HLA-DR, human glycoprotein of the MHC-II group expressed on the surface of immunocompetent cells, a significant increase in the number of reactive microglia was found in the substantia nigra of PD patients. Interestingly, reactive microglia was also found to be enhanced in the hippocampus of PD patients who also presented dementia [9].

Neuronal death in PD precedes the development of motor symptoms by many years. The mechanisms underlying the progressive neurodegeneration in PD are still elusive and the discovery of the active or main driving force is of paramount importance in the search of effective therapeutic strategies. Neuroinflammation has been proposed to actively participate in PD onset and progression. An acute insult to the central nervous system (CNS) triggers microglial activation, leading to a series of changes in microglia, notably in shape, increased proliferation, and production of inflammatory mediators that can stimulate the recruitment of peripheral leukocytes to the CNS. This inflammatory process can be regarded as beneficial for neuronal tissue, since it promotes clearance of cell debris and secretion of neurotrophic factors. Conversely, inflammatory mediators do not only modulate immune cells but also act on neurons and contributing to neurodegeneration. Neuronal death further activates inflammatory mechanisms, resulting in a vicious cycle of inflammation and neuronal death. Therefore, inflammatory responses, although essential for tissue homeostasis, can contribute to neuronal injury when it is not controlled and/or chronic (Figure 1). As neural tissues have a restricted cell renewal and regenerative capacity, CNS is extremely vulnerable to uncontrolled immune and inflammatory processes [10]. Dopaminergic neurons from substantia nigra are particularly vulnerable to microglial-mediated neurotoxicity [11].

Banati et al. demonstrated higher microglial activation in the substantia nigra of patients with PD as indicated by increased expression of CR3/43 and EBM11, markers for activated microglia [12]. The number of activated microglia (MHC-II, ICAM-1, and LFA-1 positive cells) in the substantia nigra and putamen of PD patients also increased in parallel with neuronal degeneration in those regions. Moreover, microglial activation persisted regardless of the presence or absence of Lewy bodies and was frequently associated with damaged neurons and neuritis [13]. The lack of reactive astrocytes in autopsies of the substantia nigra and putamen from PD patients contrasts with the response (with reactive astrocytes and microglia) typically found in other neurological disorders (e.g., seizures), supporting the hypothesis that the inflammatory process in PD is a unique phenomenon [14]. Autopsy brain tissue acquired from substantia nigra and basal ganglia of PD patients demonstrated that *α*-synuclein is present in regions of brain where microglial activation is known to be also present. Furthermore, an* in vitro* stimulation of murine microglia with aggregated and nitrated *α*-synuclein shift microglial morphology to an amoeboid shape and elicited dopaminergic neurotoxicity. The mechanism by which *α*-synuclein activates and alters the function of microglia in PD is not yet clear, although evidence from genomic and proteomic assays has supported a role for the transcript factor nuclear factor-kappa B [15]. Taken together these studies provide evidence supporting CNS immune resident cells role in PD. Whether microglia activation is a secondary event following the ongoing neurodegeneration or a primary inducer of the disease remains to be defined.

### 2.2. Central Nervous System Inflammatory Mediators in PD

Over the past decades, apart from microglia activation, a growing body of clinical and experimental research has been supporting a role for oxidative stress and inflammatory mediators (cytokines and chemokines), events potentially associated with microglial reaction, in PD [13, 16–18]. For instance, higher expression of the chemokine receptor CXCR4 and of its natural ligand CXCL12 was found in dopaminergic neurons of the substantia nigra of patients with PD, and this was associated with an increase in microglial activation [18]. CXCL12/CXCR4 signaling can induce neurotoxic events, including activation of caspase-3, leading to neuronal death by apoptosis. Negative effects on the CNS mediated by CXCL12 could be induced through a direct action on dopaminergic neurons expressing CXCR4 or the release of cytokines from microglia [18, 19]. A direct link between CXCL12/CXCR4 upregulation and loss of dopaminergic neurons was provided in an animal model of degeneration of the nigrostriatal system following 1-methyl-4-phenyl-1,2,3,6-tetrahydropyridine (MPTP) administration, a well-recognized model of PD [18]. The presence of activated microglia expressing the inflammatory cytokines interleukin- (IL-) 6 and tumor necrosis factor- (TNF-) *α*, as well as enzymes associated with inflammation, such as inducible isoform of nitric oxide synthase (iNOS) and cyclooxygenase-2 (COX-2) was also evidenced by immunohistochemistry assays in* post-mortem* brain tissue from PD patients [13, 16, 20]. A previous study demonstrated an enhancement in the inflammatory cytokine IL-1*β* 511 polymorphism from DNA extracted from brain tissues of PD patients [21]. Similar findings were reported for IL-6 and TNF-*α* using peripheral tissue samples (i.e., blood or buccal samples), indicating polymorphisms in these cytokines as risk factors of PD [22–24].

Upregulation of inflammatory mediators involved in apoptotic cell death through TNF-*α*-induced signaling pathway, including caspase-1, caspase-3, and TNF receptor R1 (TNF-R1 or p55), was identified in the substantia nigra from Parkinsonian patients, indicating the occurrence of a proapoptotic environment in PD [25]. Neutralization of soluble TNF signaling* in vivo* with dominant-negative TNF inhibitor XENP345 (a PEGylated version of the TNF variant A145R/I97T) abrogated in 50% the dopaminergic neuronal degeneration in an experimental model of PD induced by striatal injection of the oxidative neurotoxin 6-hydroxydopamine (6-OHDA) [17]. A more recent study demonstrated that long-lasting TNF-*α* expression induced by the injection of an adenovector expressing soluble mouse TNF-*α* (AdTNF*α*) directly in the substantia nigra of adult rats leads to dopaminergic neuronal death, motor symptoms, and microglia activation associated with recruitment of peripheral monocytes [26]. Similar findings were reported following chronic expression of IL-1*β* induced by 60 days administration of a recombinant adenovirus expressing IL-1*β* in the substantia nigra of adult rats [27]. Interestingly, alterations in mRNA expression of mediators of the immune response during PD, including members of the complement system, colony stimulating factors, Toll family, and cytokines, seem to occur in a brain region-dependent manner. For instance, a downregulation in the mRNA expression of tumor necrosis factor related protein 7 (C1QTNF7), a member of the complement system, was found in the substantia nigra whereas an upregulation was observed in the putamen of PD patients at the same stage of the disease. Immunohistochemistry also reveals the expression of cytokines, including IL-6 and TNF-*α*, by microglia and neurons in the PD substantia nigra and frontal cortex [28]. Active NF*κ*B is localized in the nucleus of subpopulations of neurons and glial cells mainly in substantia nigra and less frequently in putamen and cerebral cortex [28]. Altogether, these studies suggest an involvement of inflammation, in particular related to CNS resident immune cells activation, in the degeneration of dopaminergic neurons associated with PD.

Cerebrospinal fluid (CSF) mirrors metabolic and pathological states of the CNS more directly than any other body fluid. Therefore, CSF is a good source for neuroinflammation evaluation and PD biomarker discovery since it is more accessible than brain tissue and less costly than imaging [29]. In this regard, studies have evaluated levels of inflammatory markers in the CSF of PD patients. Increased levels of IL-1*β* and IL-6 were found in the CSF of PD patients [30]. Corroborating these findings, concentrations of IL-2 and IL-6 were higher in ventricular CSF from PD patients in comparison with control subjects. In addition, concentrations of IL-1*β*, IL-2, IL-4, and transforming growth factor- (TGF-) *α* in ventricular CSF were higher in juvenile PD patients (PD manifesting clinically bellow the age of 40) than those in controls [31]. Free TGF-*β*1 and total TGF-*β*2 levels were elevated in* post-mortem* ventricular CSF of patients with PD in comparison with age and gender-matched controls [32]. However, one study failed to find significant differences in CSF levels of the inflammatory markers C-reactive protein (CRP), IL-6, TNF-*α*, eotaxin, interferon gamma-induced protein 10 (IP-10), monocyte chemotactic protein 1 (MCP-1), and macrophage inflammatory protein- (MIP-) 1*β* from PD patients in comparison with a reference group [33].

Using a highly sensitive Luminex assay, one study assessed a series of CSF molecules in PD, Alzheimer's disease (AD), multiple system atrophy (MSA) patients, and healthy controls: total tau, phosphorylated tau, amyloid beta peptide 1–42 [A*β*(1–42)], Flt3 ligand, and fractalkine. CSF levels of Flt3 clearly differentiated PD from MSA, a disease that clinically overlaps with PD, with excellent sensitivity (99%) and specificity (95%). In addition, CSF fractalkine/A*β*(1–42) ratio positively correlated with PD severity and PD progression. Flt3 ligand and fractalkine are inflammatory markers possibly related to PD [29].

### 2.3. Insights from the Genetic Leucine-Rich Repeat Kinase 2 (LRRK2) Model of Neuroinflammation Associated with PD

Animal models of PD have become valuable tools to the understanding of its pathophysiology, regardless of their limitations in mimicking all features of the human disease. Neurotoxin-based animal models (6-OHDA and MPTP), referred to as pathogenic models, have largely been used to induce selective neuronal death in both* in vitro* and* in vivo* studies. Currently, genetic-based models (or etiologic models), such as those related to mutations in the Leucine-rich repeat kinase 2 (LRRK2) gene, have opened new directions of investigation of molecular and cellular mechanisms underlying PD pathogenesis [34, 35].

Fine-mapping, gene expression, and splicing analysis from human* post-mortem* brain tissues have supported a role for LRRK2 gene in PD. There is convincing evidence for a common variant PD association located outside of the LRRK2 protein coding region (rs117762348) [36]. In this scenario, it has been shown that activated myeloid lineage cells, including macrophages and microglia, presented high levels of LRRK2, suggesting an involvement of this gene in the neuroinflammation associated with PD [37, 38]. An elegant study demonstrated that *α*-synuclein overexpression in rats' substantia nigra induced LRRK2 expression in activated microglial cells, and this correlated with a high expression of iNOS, known to be involved in PD [39]. LRRK2 knockout rats are protected from dopaminergic neurodegeneration elicited by *α*-synuclein overexpression or intracranial administration of lipopolysaccharide (LPS). Neuroprotection observed in the absence of LRRK2 was associated with reduction in proinflammatory CD68-positive myeloid cells in the substantia nigra, indicating an involvement of LRRK2 in conditions where neuroinflammation may underlie neuronal dysfunction and degeneration such as PD [39].

### 2.4. Positron Emission Tomography (PET) as a Diagnostic Tool for Neuroinflammation Related to PD

Positron emission tomography (PET) is a noninvasive functional imaging technique that detects gamma rays emitted by a positron-emitting radionuclide (tracer) which is introduced into the body on a biologically active molecule [40].

The isoquinoline carboxamide PK11195 is currently the most widely used ligand for the translocator protein 18 kDa (TSPO, also known as peripheral benzodiazepine receptor). TSPO is a marker of microglial activation and has been used to assess and quantify the dynamics of activated microglia in neurodegenerative diseases, including PD. [^11^C]PK11195 is used in PET studies for imaging brain inflammation* in vivo* [41]. PET studies using [^11^C]PK11195 demonstrated increased binding potential values (parameter that mixes receptor density with ligand affinity) in the midbrain as well as in the pons, basal ganglia, and frontal and temporal cortices in PD, indicating an anatomically widespread distribution of microglial activation, possibly associated with the pathological process of PD [42, 43]. Longitudinal analysis of these patients revealed stable [^11^C]PK11195 binding potential values, indicative of early activation of microglia in PD pathogenesis [43]. However, [^11^C]PK11195 tracer cannot distinguish between microglial protective or damaging profile. To overcome this, a PET tracer for the dopamine-transporter (DAT), [^11^C]CFT, has been used in conjunction with [^11^C]PK11195 in order to further investigate microglial activation in parallel with the viability of the presynaptic dopaminergic neurons. Midbrain [^11^C]PK11195 binding potential levels were inversely correlated with [^11^C]CFT binding potential values in the putamen and positively correlated with the severity of motor symptoms, suggesting that neuroinflammation associated with microglial activation might contribute to the progression of the disease [44]. PET imaging has also been employed to investigate* in vivo* potential therapeutic strategies for PD. For instance, [^11^C]PK11195 PET was used to evaluate the ability of COX-2 inhibition with celecoxib to reduce neuroinflammation in PD patients. Patients showed higher putamen and midbrain binding potential in comparison with controls, but considerable overlap was seen between groups, and differences were not statistically significant. This prevented reliable assessment of the changes in the [^11^C]PK11195 uptake by celecoxib treatment [45]. In a rat model of PD induced by intrastriatal administration of 6-OHDA, PET imaging revealed that the COX-2 inhibitor celecoxib decreased microglial activation and prevented dopaminergic neuron degeneration [46]. A study conducted by Edison et al. demonstrated by PET analysis that both PD patients with or without dementia presented significant microglial activation in cortical brain regions, suggesting that neuroinflammation could be an early phenomenon in PD, persisting as the disease progress [47].

## 3. Peripheral Immune Response in PD

### 3.1. Peripheral Immune Biomarkers

A great body of evidence regarding peripheral inflammatory/immune markers has supported the hypothesis of inflammation involvement in PD. Studies of cytokines in serum or plasma have revealed increased levels of proinflammatory cytokines such as TNF-*α* [48, 49] and its soluble receptors sTNFR1 [50, 51] and sTNFR2 [51] and IL-1*β* [52] in PD patients in comparison with matched controls. Increased serum levels of macrophage migration inhibitory factor (MIF) were found in PD patients in comparison with healthy subjects [53]. Also the levels of IL-2 [54, 55], interferon (IFN)-*γ* [54], IL-6 [49, 54, 56, 57], and the anti-inflammatory cytokine IL-10 were described to be increased in PD [54, 58]. IL-6 plasma concentration was prospectively associated with an increased risk of developing PD [58]. In contrast, some authors failed to show significant alterations in cytokine levels in PD. Peripheral levels of the cytokines IL1-*α*, IL-6, TNF-*α* [50, 52, 59, 60], IFN-*γ*, IL-2, IL-4, IL-10 [61], and IL-12 [62] were similar in PD patients and age- and gender-matched controls. Circulating levels of the chemokines MIP-1*α*, IL-8 [63], eotaxin, eotaxin-2, IP-10 [63, 64], and MCP-1 [64] did not differ between PD patients and controls. These controversial findings could be explained, at least in part, by methodological differences among the studies, including heterogeneous PD samples and different techniques to measure the molecules.

Apart from serum/plasma studies, the concentration of cytokines produced by peripheral cells* in vitro* has been assessed in PD. Both basal production and LPS-induced production of MCP-1, MIP-1*α*, IL-8, IFN-*γ*, IL-1*β*, and TNF-*α* were significantly higher in PD patients compared with control subjects [65]. Conversely, the secretion of IL-2 by peripheral blood mononuclear cells (PBMC) after mitogenic stimulation was decreased in PD patients in comparison with controls, whereas IL-6, IFN-*α*, IFN-*γ*, and sIL-2R levels were comparable in both groups [66].

Several case reports of IFN-*α*-induced Parkinsonism in chronic hepatitis patients further corroborate the hypothesis of the role played by peripheral inflammation in PD pathogenesis [67–69]. The relationship between PD and systemic infections also supports this hypothesis. For instance, in a population-based case-control study in British Columbia, Canada, severe influenza infection was associated with PD, although this effect was attenuated when cases were restricted to those occurring ten or more years before diagnosis (Figure 1) [70].

### 3.2. Peripheral Immune Cells

Studies have also described changes in the percentage of peripheral blood immune cells in PD, such as lower total lymphocyte counts in comparison with controls [71–73]. Reduction in the total number of lymphocytes may result from the decrease in the percentage of T (CD3+) and B (CD19+) cells in PD patients. Changes in CD3+ cells were associated with a reduction in T helper (Th, CD4+) lymphocytes, while T cytotoxic (CD8+) cells increased or remained unchanged [71–74]. Lower number of CD4+ cells could be explained by the fact that in PD these cells presented both increased spontaneous apoptosis and activation-induced apoptosis [75].

Not only the percentage of circulating immune cells but also their activation profile must be taken into account when evaluating immune parameters. One study showed that the number of “naïve” (CD4+CD45RA+) and memory helper (CD4+CD29+) T cells was decreased, while the number of activated (CD4+CD25+) T cells was increased in PD [71]. In addition, impaired ability of regulatory T cells (Treg) to suppress effector T cell function has been described in PD patients [73]. Increased oxidative stress may also be associated with changes in lymphocyte profile in PD, since both whole cell and mitochondrial reactive oxygen species (ROS) in peripheral blood mononuclear cells are increased in PD [76].

Some studies have reported similar percentages of CD3+ lymphocytes in PD patients and control subjects [52, 72]. T helper lymphocytes (CD4+) were decreased, while CD8+ cell counting increased in PD [72].

There is evidence of higher percentage of natural killer (NK) cells in peripheral blood of PD patients compared to controls, and this increase has been associated with disease severity and progression [52, 61, 77]. Despite increased number of NK cells in PD, their activity seems to be unchanged in PD [61, 77].

### 3.3. The Concomitant Effect of Inflammaging

PD is unequivocally an age-related disorder. Aging is a complex process accompanied by many physiological changes, notably in the immune system. Aging results in an increase in systemic levels of inflammatory markers, indicating the presence of subtle chronic inflammation, a phenomenon known as inflammaging. Chronic inflammation damages cells of the brain, heart, arterial walls, and other body structures, contributing to the onset and progression of a broad spectrum of degenerative diseases of aging, including heart disease, rheumatoid arthritis, AD, and PD. Inflammation generates oxidative stress, which might contribute to neuronal death in diseases such as AD, PD, and amyotrophic lateral sclerosis (ALS) (Figure 1) [78].

## 4. GWAS Studies: Further Evidence for a Role of Inflammation in PD

Genome-wide association studies (GWAS) have also identified genetic markers that link PD and inflammation. Hamza et al. detected an association between PD and the human leukocyte antigen (HLA) region (chromosome 6p21.3), finding replicated in two datasets with Caucasians (North-American of European ancestry). Associations were particularly strong for individuals with sporadic and late-onset PD and men. The variant most strongly associated with PD was rs3129882 in intron 1 of HLA-DRA [79]. The protein chains are encoded by the closely linked HLA-DRA and HLA-DRB form the class II HLA-DR antigens that are expressed by antigen-presenting cells, including microglia in the brain, and interact with T-cell receptors [79]. This result is in line with PD specific overexpression of HLA-DR antigens in substantia nigra [9]. One study has also confirmed HLA region as PD risk locus among the Dutch population [80].

One GWAS was conducted to identify common genetic variants associated with motor and cognitive outcomes in PD. The single nucleotide polymorphisms (SNP) rs10958605 (C8orf4 gene) and rs6482992 (CLRN3 gene) were associated with motor and cognitive outcomes, respectively. The encoded protein by C8orf4 gene may play a role in the NF-*κ*B and ERK1/2 signaling pathways, highlighting inflammation as a possible pathogenesis mechanism for progression in PD [81].

A recent meta-analysis has identified four loci, including the HLA region, that contain a secondary independent risk variant for PD that exerts an effect independently of the primary risk allele [82].

Genetic factors may also be essential in determining an individual's susceptibility to inflammation-induced nigral dopaminergic neuronal cell death (Figure 1) [83].

## 5. Immune Changes Induced by Antiparkinsonian Drugs 

Long-term treatment with antiparkinsonian drugs may result in changes in immune system. For example, treatment with amantadine, originally established as an antiviral drug, was associated with an increase of the CD4 : CD8 ratio [84]. Treatment with amantadine has been described to increase IL-2 levels [85, 86]. The same was not observed in patients in use of levodopa as monotherapy [85]. Levodopa therapy induced changes in T lymphocytes proteome [87]. Levodopa-treated patients showed significantly higher IL-15 and RANTES circulating levels in comparison with healthy controls and higher, but not statistically significant levels, with respect to untreated patients [88].

In order to evaluate a putative immunomodulatory role of levodopa, PBMC of PD patients and controls were incubated* in vitro* with the drug. Levodopa caused an inhibition of mitogen-induced proliferation, stimulation of IL-6, and TNF-*α* production, whereas the secretion of IL-1*β* and IL-2 was not affected in both groups [89].

## 6. Nonsteroidal Anti-Inflammatory Drugs (NSAIDs) Use and Risk of PD

Based on the hypothesis that neuroinflammation is involved in PD pathophysiology, epidemiological studies have evaluated nonsteroidal anti-inflammatory drugs (NSAIDs) use and risk of PD. The first study conducted with this purpose was a prospective cohort in which the regular use of NSAIDs, but not aspirin, was associated with a delay or prevention of PD onset [90]. The same research group later investigated whether NSAIDs use was associated with a lower risk for PD in a large cohort with more detailed information on different types of NSAIDs. They found no association between the use of aspirin, other NSAIDs, or acetaminophen and PD risk. Interestingly, PD risk was lower among ibuprofen users than nonusers, suggesting that ibuprofen use may delay or prevent the onset of PD [91]. In line with these results, a prospective study revealed that ibuprofen users had a significantly lower PD risk than nonusers, even when adjusting for age, smoking, caffeine consumption, and other covariates. The same effect was not observed for aspirin, other NSAIDs, or acetaminophen [92]. Since only the use of ibuprofen, but not other NSAIDs, was associated with lower PD risk, some specific effects of ibuprofen may be important. In fact, an earlier study examined the effects of NSAIDs drugs on cultured primary rat embryonic neurons from mesencephalon, the area primarily affected in PD. Ibuprofen protected both dopaminergic neurons and other neurons against glutamate toxicity. In addition, ibuprofen alone increased the relative number of dopaminergic neurons by 47% [93].

In contrast with the above mentioned studies, a population-based study described a decreased risk of PD among regular aspirin users. A stronger protective effect was observed for regular nonaspirin NSAIDs users. It is noteworthy that the aspirin effect differed by gender, showing a protective effect only in women, especially among long-term regular users [94]. The most recent study supporting the association between NSAIDs and reduced PD risk was conducted in 2008. NSAIDs use was described to significantly reduce PD risk in 20% to 30%. The effect of the combination of NSAIDs use and smoking and coffee consumption was also evaluated. People who were at the highest exposure to smoking and coffee and used NSAIDs had an estimated 87% reduction in PD risk. As properly stated by the authors, whether this finding reflects true biological protection needs to be further investigated [95].

There are studies that failed to show any association between NSAIDs use and PD [96–102]. The discrepant results may be due to different methods used to conduct the investigations, especially how authors collected data about NSAID use (medical records, self-report, pharmacy databases, etc.) and the evaluated population.

Several case-control studies have been performed to examine the association between NSAIDs use and PD risk. Given the discrepancy in results, meta-analysis is of great value to better define this association. A meta-analysis with this purpose concluded that NSAIDs do not seem to modify the risk of PD. However, ibuprofen may have a mild protective effect in lowering the risk of PD [103]. Another meta-analysis estimated an overall reduction in 15% in PD incidence among users of nonaspirin NSAIDS, with a similar effect observed for ibuprofen use. The protective effect of nonaspirin NSAIDs was more pronounced among regular and long-term users. No protective effect was observed for aspirin or acetaminophen [104]. In conclusion, there is evidence for a protective effect of nonaspirin NSAIDs use in relation to PD, which is consistent with the neuroinflammatory hypothesis for PD pathogenesis.

In this scenario, among several studies evaluating anti-inflammatory strategies in animal models of PD, one is noteworthy. The nitric oxide (NO)-NSAID HCT1026 [2-fluoro-*α*-methyl(1,1′-biphenyl)-4-acetic-4-(nitrooxy)butyl ester], NO-donating flurbiprofen, is an anti-inflammatory agent obtained by derivatization of conventional NSAIDs with a NO-donating moiety which strongly reduces their untoward side effects without altering the anti-inflammatory effectiveness. Oral treatment with HCT1026 showed a safe profile and a significant efficacy in counteracting MPTP-induced dopaminergic neurotoxicity, motor impairment, and microglia activation in aging mice [105], providing a promising approach towards the development of effective pharmacological neuroprotective strategies against PD.

## 7. Autoimmunity and Immune-Based Therapies in PD

PD has been associated with autoimmunity. Juvenile Parkinsonism has been reported as a manifestation of systemic lupus erythematosus [106]. Anecdotal reports tried to establish an association between PD and rheumatoid arthritis [107, 108]. Antibodies against dopaminergic neurons were demonstrated in the serum of a patient with a complex autoimmune disorder and rapidly progressing PD [109]. One study reported significantly higher antibody levels towards monomeric *α*-synuclein in the sera of PD patients compared to controls, and their levels decreased with PD progression. According to these authors, this possibly indicates a protective role of autoimmunity in maintaining body homeostasis and clearing protein species whose imbalance may lead to misfolded protein aggregation [110].

All currently available treatments for PD are of only symptomatic benefit, and a pharmacological strategy with disease-modifying effect is highly needed. In this context, immune-based therapies have been proposed for PD treatment. The first strategy was based on immunotherapy against aggregated forms of *α*-synuclein. Transgenic mice displaying abnormal accumulation of human *α*-synuclein and *α*-synuclein-immunoreactive inclusion-like structures in the brain were vaccinated with human *α*-synuclein. There was decreased accumulation of aggregated *α*-synuclein in neuronal cell bodies and synapses, and, as a consequence, reduced neurodegeneration. Similar effects were observed with an exogenously applied FITC-tagged *α*-synuclein antibody [111]. The same work group showed that passive immunization with a monoclonal *α*-synuclein antibody (9E4) against the C-terminus *α*-synuclein reduced the accumulation of calpain-cleaved *α*-synuclein in axons and synapses in the *α*-synuclein transgenic mice. In addition, 9E4 was able to cross the blood brain barrier into the CNS, to bind to cells displaying *α*-synuclein accumulation and to promote *α*-synuclein clearance via the lysosomal pathway [112].

Studies on AD have provided valuable information about immunotherapy in neurodegenerative disorders. Immunotherapy against the *β*-amyloid peptide in AD showed that approaches targeting cerebral proteins can be applied to humans with relative safety. Neuropathological examination showed the clearance of amyloid plaques in brains of AN1792-vaccinated AD patients. Nonetheless, relevant issues must be considered. For instance, T cell responses specific for cerebral antigens need to be avoided. Another important issue is to define which patient should be vaccinated. Disease-modifying approaches are more effective when applied in the early stage of the disease, when diagnosis is not established yet [113].

AFFITOPE PD01, the most promising vaccine developed for PD so far, entered clinical trials and therefore represents the first PD vaccine to be tested clinically. AFFITOPE PD01 has been developed to induce antibodies recognizing *α*-synuclein but sparing the family member *β*-synuclein, which has neuroprotective properties [113].

Immune stimulation in the periphery may also provide a new strategy to halt PD progression. In addition to studies on immunotherapy against aggregated forms of *α*-synuclein, one study described the neuroprotective effects of Bacillus Calmette-Guérin (BCG) vaccination in the MPTP mouse model of PD. BCG vaccination had a significant beneficial effect on both striatal dopamine content and DAT ligand binding levels. BCG vaccination prevented the increase in the number of activated microglia in the substantia nigra induced by the MPTP, suggesting that general immune stimulation in the periphery can limit CNS microglia response to a neuronal insult [114].

## 8. Conclusion

We reviewed the evidence regarding the contribution of immune dysfunction and/or inflammation in PD, including microglial activation and brain and peripheral levels of immune mediators. Assessment of these biomarkers may contribute to the development of diagnostic and prognostic tools in PD. In addition, the protective role of NSAIDs further supports the neuroinflammation hypothesis in PD.

## Figures and Tables

**Table 1:** Evidence regarding the contribution of immune dysfunction and/or inflammation in Parkinson's disease.

Evidence	Source	Results	Reference
CNS inflammation	Human brain	Significant increase in the number of reactive microglia in the substantia nigra of PD patients.	[9, 12]
		Coexistence of *α*-synuclein and activated microglia.	[15]
		Higher expression/increased levels of inflammatory mediators in PD brains.	[13, 16–18]
	Human CSF samples	Increased levels of IL-1*β*, IL-2, IL-4, IL-6, TGF-*α*, free TGF-*β*1, and total TGF-*β*2 in the CSF of PD patients.	[30–32]

Peripheral inflammation	Serum/plasma samples	Increased levels of IFN-*γ*, IL-1*β*, IL-2, IL-3, IL-10, MIF, TNF-*α*, and its soluble receptors sTNFR1 and sTNFR2 in PD patients samples.	[48–58]
	Supernatants from cell cultures	MCP-1, MIP-1*α*, IL-8, IFN-*γ*, IL-1*β*, and TNF-*α* levels were significantly higher in PD patients.	[65]
	Blood leukocytes	PD patients exhibited lower total lymphocyte counts; decrease in the percentage of T (CD3+) and B (CD19+) cells and reduction in T helper (Th, CD4+) lymphocytes; higher percentage of NK cells.	[52, 61, 71–74, 77]

Genetic evidence	DNA extracted from brain, blood, or buccal samples	Enhancement in IL-1*β* 511, IL-6, and TNF-*α* polymorphisms.	[21–24]

Epidemiological evidence	Clinical and population-based studies	NSAIDs use was associated with a lower risk for PD.	[91, 92, 94, 95, 104].
		IFN-*α*-induced Parkinsonism in chronic hepatitis [67–69].	[67–69]
		The relationship between PD and systemic infections (severe influenza).	[70]

CSF: cerebrospinal fluid; CNS: central nervous system; IFN: interferon, IL: interleukin; MIF: migration inhibitory factor; MCP: monocyte chemotactic protein; MIP: macrophage inflammatory protein; NSAIDs: nonsteroidal anti-inflammatory drugs; PD: Parkinson's disease; TGF: transforming growth factor; TNF: tumor necrosis factor; sTNFR: TNF soluble receptor.
